# The NAC-type transcription factor CaNAC46 regulates the salt and drought tolerance of transgenic *Arabidopsis thaliana*

**DOI:** 10.1186/s12870-020-02764-y

**Published:** 2021-01-06

**Authors:** Jing Ma, Li-yue Wang, Jia-xi Dai, Ying Wang, Duo Lin

**Affiliations:** College of Horticulture, Qingdao Agricultural University, Key Laboratory of Horticultural Plant Genetic Improvement and Breeding of Qingdao, 700 Changcheng Road, Qingdao, 266109 China

**Keywords:** Pepper, NAC transcription factor, Stress tolerance, Transgenic plants

## Abstract

**Background:**

The NAC (NAM, ATAF1/ATAF2, and CUC2) transcription factors belong to a large family of plant-specific transcription factors in monocot and dicot species. These transcription factors regulate the expression of stress tolerance-related genes that protect plants from various abiotic stresses, including drought, salinity, and low temperatures.

**Results:**

In this study, we identified the *CaNAC46* transcription factor gene in *Capsicum annuum*. Its open reading frame was revealed to comprise 921 bp, encoding a protein consisting of 306 amino acids, with an isoelectric point of 6.96. A phylogenetic analysis indicated that CaNAC46 belongs to the ATAF subfamily. The expression of *CaNAC46* was induced by heat, cold, high salt, drought, abscisic acid, salicylic acid, and methyl jasmonate treatments. Thus, CaNAC46 may be important for the resistance of dry pepper to abiotic stresses. A subcellular localization analysis confirmed that CaNAC46 is localized in the nucleus. The overexpression of *CaNAC46* improved the tolerance of transgenic *Arabidopsis thaliana* plants to drought and salt stresses. The *CaNAC46*-overexpressing lines had longer roots and more lateral roots than wild-type lines under prolonged drought and high salt stress conditions. Additionally, CaNAC46 affected the accumulation of reactive oxygen species (ROS). Moreover, CaNAC46 promoted the expression of *SOD*, *POD*, *RD29B*, *RD20*, *LDB18*, *ABI*, *IAA4*, and *P5CS*. The malondialdehyde contents were higher in *TRV2-CaNAC46* lines than in wild-type plants in response to drought and salt stresses. Furthermore, the expression levels of stress-responsive genes, such as *ABA2*, *P5CS*, *DREB*, *RD22*, *CAT*, and *POD*, were down-regulated in *TRV2-CaNAC46* plants.

**Conclusions:**

Under saline and drought conditions, CaNAC46 is a positive regulator that activates ROS-scavenging enzymes and enhances root formation. The results of our study indicate CaNAC46 is a transcriptional regulator responsible for salinity and drought tolerance and suggest the abiotic stress-related gene regulatory mechanisms controlling this NAC transcription factor are conserved between *A. thaliana* and pepper.

**Supplementary Information:**

The online version contains supplementary material available at 10.1186/s12870-020-02764-y.

## Background

Pepper (*Capsicum annuum*; family: Solanaceae) is a popular vegetable crop cultivated worldwide. China is the largest producer and consumer of chili peppers, with approximately 40% of the global chili cultivation area [1]. In addition to being widely used as a condiment, dry peppers are also used in the medical and military industries [2]. However, there are some environmental conditions, such as salinization and drought, that adversely affect the main dry pepper-producing areas.

Abiotic stresses, including drought, high-salinity, and temperature extremes, are the main factors responsible for decreased dry pepper quality [3]. To protect against the detrimental effects of environmental stresses, plants have gradually evolved appropriate defense mechanisms, in which the regulation of gene expression plays an important role. Transcription factors are critical regulators of abiotic stress tolerance [4]. In plants, the large NAC (NAM, ATAF1/ATAF2, and CUC2) transcription factor family is involved in abiotic stress responses [5]. The N-terminal of NAC transcription factors is highly conserved, in contrast to the variable C-terminal [6]. This transcription factor family has recently been extensively studied in *Arabidopsis thaliana*, rice, tobacco, and soybean [7–10]. There has also been increasing interest in these transcription factors in pepper crops [11, 12]. A genome-wide analysis of 104 NAC transcription factors in hot pepper and an expression analysis of 22 NAC transcription factor genes revealed diverse responses to various stresses [13]. As a transcriptional activator of *CaPLD4*, CaNAC1 helps degrade phospholipids in green pepper in response to low temperatures [11]. The pepper *CaNAC2* transcription level is reportedly considerably up-regulated by an abiotic stress treatment, but is down-regulated by osmotic stress and a salicylic acid (SA) treatment [14].

The *CaNAC46* gene was identified during a search of a pepper cDNA library with *A. thaliana AtNAC2*/*ATAF1*/*ANAC002* (At1g01720) as the query [14]. The CaNAC46 transcription factor helps regulate responses to abiotic stresses (e.g., drought, salinity, and low temperatures) and phytohormones [e.g., abscisic acid (ABA), methyl jasmonate (MeJA), and SA]. We demonstrated that CaNAC46 positively regulates plant tolerance to drought and salinity stresses, and developed a model for the role of CaNAC46 in the abiotic stress response system [15]. The results may provide the basis for a comprehensive analysis of the function and regulatory network of CaNAC46 related to plant stress resistance, with potential implications for the application of *CaNAC46* in future genetic engineering experiments.

## Results

### Identification of the ATAF subfamily gene *CaNAC46* in pepper

The NAC transcription factors are involved in mediating plant responses to abiotic stresses [16, 17]. The *CaNAC46* gene was isolated from *C. annuum* var. ‘Qingnong No. 2’. The encoded protein comprised 306 amino acids, with a molecular weight of 34.94 kDa and a pI of 6.96 (Additional file 1: Table S1). Additionally, the proportions of basic and acidic amino acids were similar between CaNAC46 and the homologous NAC proteins in other model plants. There were also similarities in the other examined physical and chemical characteristics between CaNAC46 and its homologs in other model plants. These results suggest that NAC transcription factors have a conserved secondary structure.

**Fig. 1:**
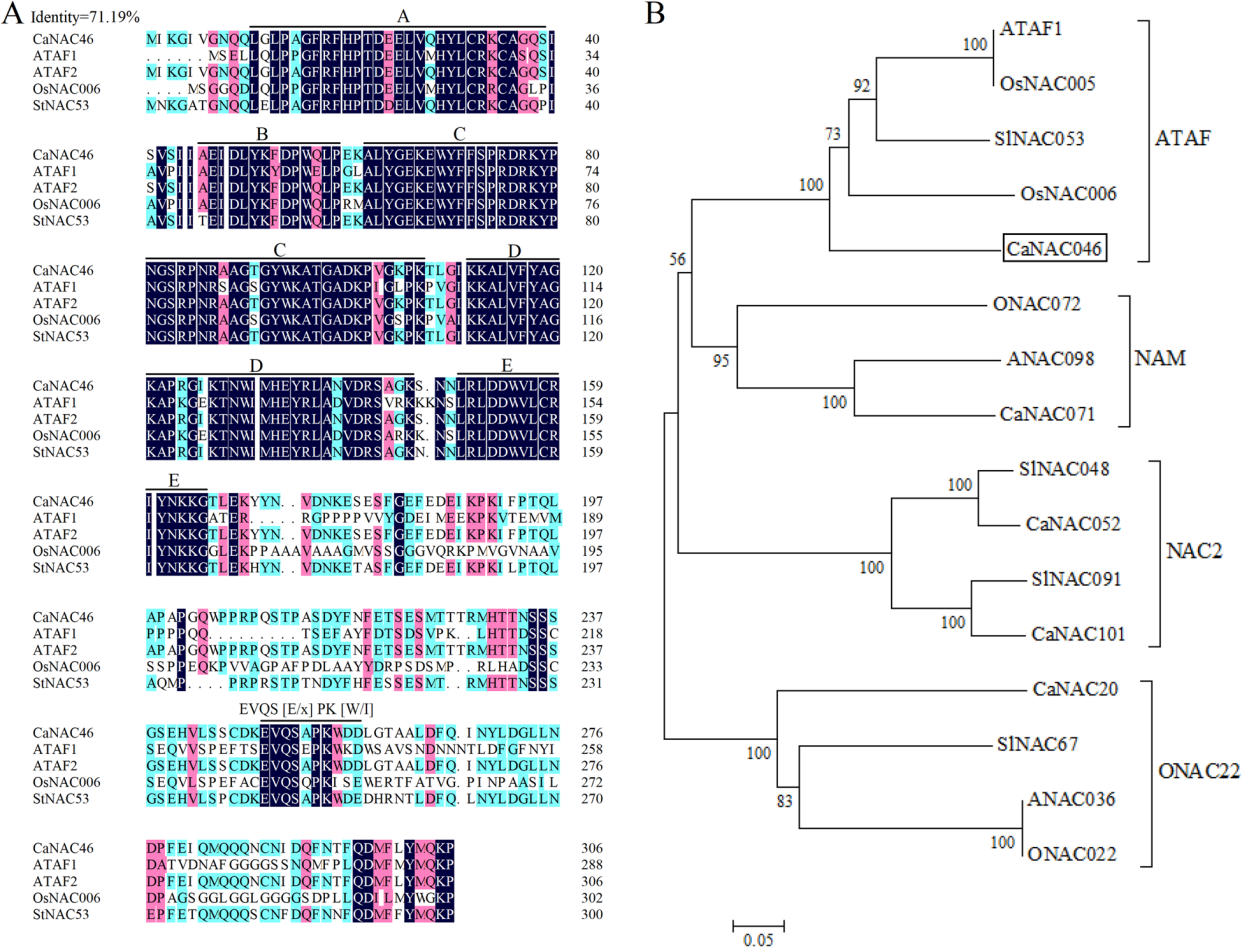
Alignment and phylogenetic analysis of CaNAC46 transcription factor. **A** Alignment of protein sequences of CaNAC46 and ATAF subfamily from other plants. **B** Phylogenetic tree of CaNAC46 and NAC members from other plant species. **At/A:** *Arabidopsis thaliana*, **Ca:** *Capsicum annuum* **Os/O:** *Oryza sativa*, **Sl:** *Solanum lycopersicum*. The protein sequences used to construct the tree were ATAF1 (At1g01720), ATAF2 (AT5G08790), ANAC036 (At2g17040), ANAC098 (At5g53950), OsNAC005 (LOC_Os11g08210), OsNAC006 (LOC_Os01g66120), ONAC072 (LOC_Os09g32260), ONAC022 (LOC_Os03g04070), SlNAC053 (Solyc06g060230), SlNAC048 (Solyc05g055470), SlNAC067 (Solyc07g053590), SlNAC091 (Solyc00g008000), StNAC53 (CAC42087.1), CaNAC52 (Solyc05g002477), CaNAC71 (Capana07g002159), CaNAC101 (Capana12g002456), CaNAC20 (Capana02g000302), CaNAC46 (Capana05g000569). The phylogenetic tree was constructed based on peptide sequences using the Neighbor-Joining method. CaNAC46 is marked by solid box

In this study, CaNAC46 was clustered with ATAF1 [18], OsNAC5 [19], and OsNAC6 [20], which belong to the ATAF subfamily (Fig. 1A). A multiple sequence alignment revealed a sequence identity of 71.19%, implying that the NAC transcription factor sequences were highly conserved. The CaNAC46 C-terminal contains the EVQS [E/x] PK [W/I] sequence, which is consistent with the sequences of typical ATAF subfamily members [3]. A phylogenetic analysis revealed that CaNAC46 is most closely related to OsNAC5, OsNAC6, and ANAC2 of the ATAF subgroup (Fig. 1B).

### CaNAC46 is localized in the nucleus

**Fig. 2:**
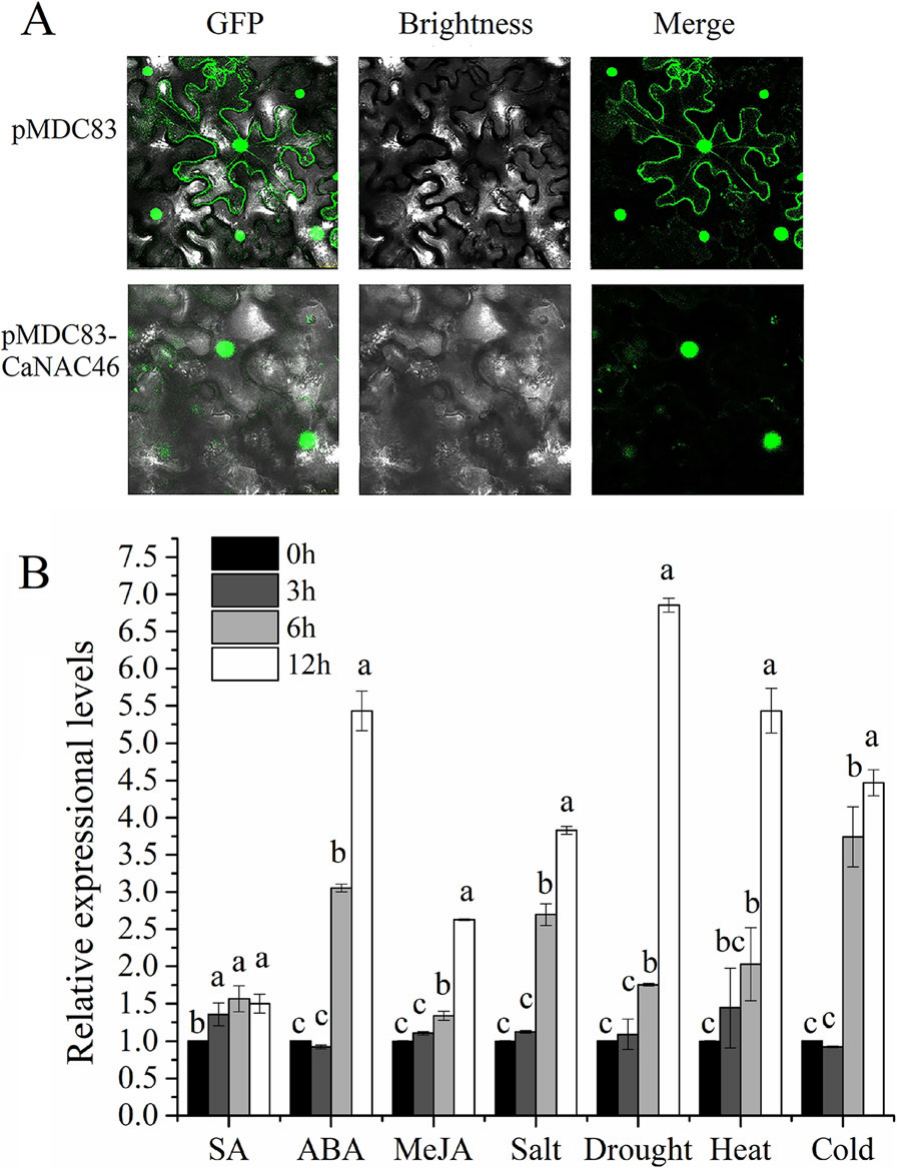
Subcellular Localization and Stress-Responsive Expression. **A** Nuclear localization of CaNAC46 in tobacco cells. **B** Expression patterns of *CaNAC46* in response to stress treatments. Superscript letters indicate significant differences. Three independent biological experiments were performed (*P* < 0.05)

An analysis of the fluorescent signals for the green fluorescent protein (GFP) alone (control) and the CaNAC46-GFP fusion protein in tobacco cells with a confocal microscope indicated CaNAC46 is a nuclear protein (Fig. 2A).

### *CaNAC46* expression is induced by abiotic stresses and phytohormones

We examined the *CaNAC46* expression level in response to various abiotic stresses over a 12-h treatment period. The expression of *CaNAC46* was significantly up-regulated following drought, salt, heat, and cold stress treatments, especially from the 6-h time-point, with peak expression levels at 12 h (Fig. 2B). Additionally, *CaNAC46* expression was also substantially induced by ABA, which is a phytohormone involved in salt and drought stress responses.

### Overexpression of *CaNAC46* enhances the drought and salinity tolerance of transgenic *Arabidopsis thaliana*

**Fig. 3:**
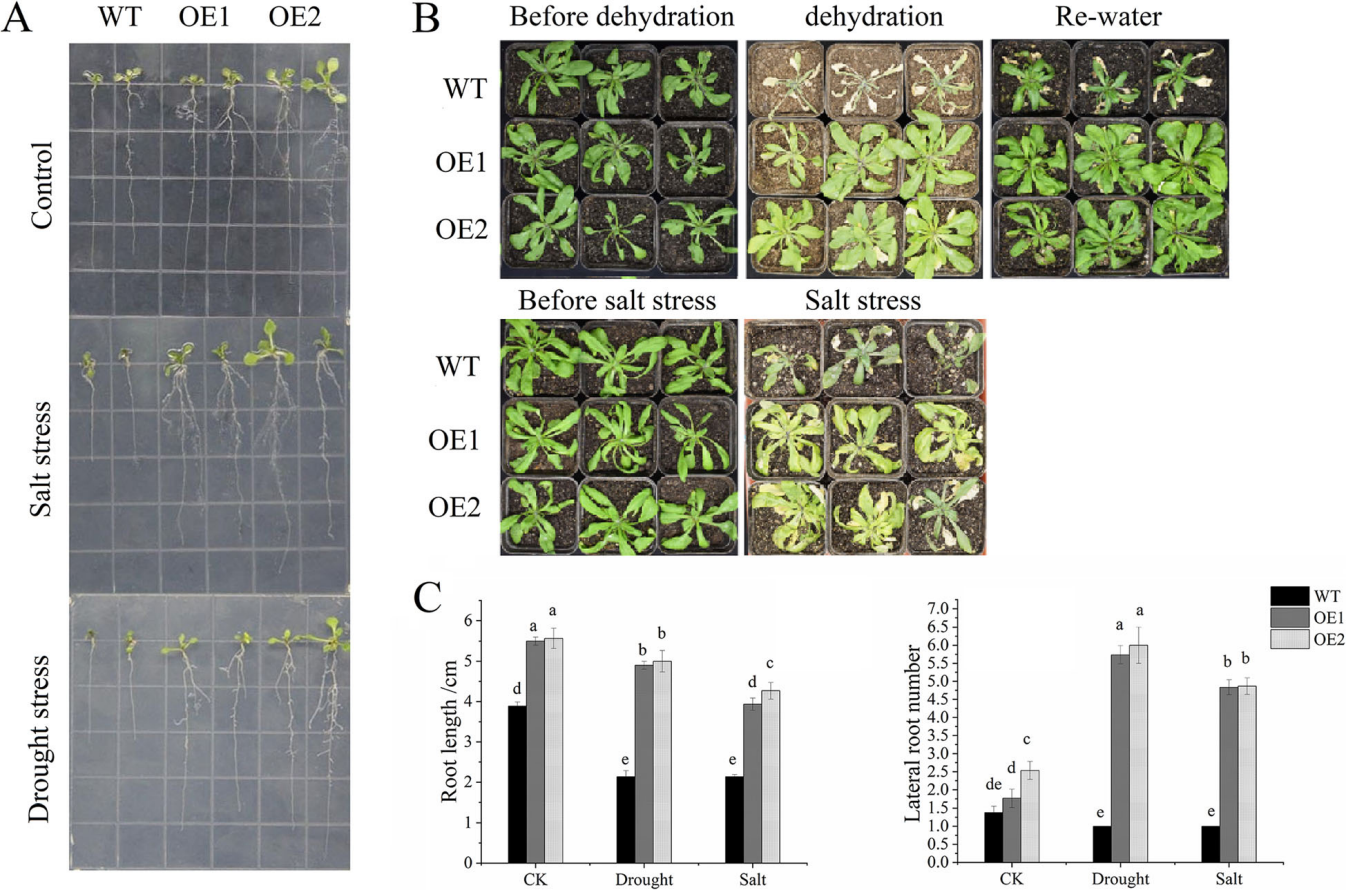
Over-expression of *CaNAC46* in *Arabidopsis* improves salt and drought stress tolerance. **A** The seedlings of transgenic and WT plants were cultured with containing 1/2 MS, 1/2 MS with 200 mM NaCl and 1/2 MS with 10% PEG6000 agar medium for 7 days. **B** Performance of 3-week-old WT, CaNAC46-OE1 and OE1 plants grown under drought stress for 3 weeks and were re-watered for 3 days (upper); 3-week-old WT, CaNAC46-OE1 and OE1 plants were watered 200 mM NaCl for 3 weeks (below). **C** The number and length of roots were counted at 1-week-old seedling stage. Three independent biological experiments were performed. Superscript letters indicate significant differences (*P* < 0.05)

The effect of *CaNAC46* overexpression on drought stress tolerance was investigated using transgenic plants treated with polyethylene glycol (PEG 6000). To assess the effect of *CaNAC46* overexpression on salt stress tolerance, wild-type (WT) and transgenic seedlings were grown on 1/2 MS agar medium supplemented with 200 mM NaCl (Fig. 3A). Under normal conditions, the germination rates of the WT and transgenic plants were approximately 95%. However, the germination rates under the simulated drought and salt stress conditions differed substantially between the WT and *CaNAC46*-overexpressing plants (Additional file 2: Figure S1).

The salinity and drought tolerance of the *CaNAC46*-overexpressing lines was further evaluated at the vegetative growth stage. Following the drought and high salt treatments, the leaves of the WT plants were severely wilted, but the leaves of the *CaNAC46*-overexpressing plants were not (Fig. 3B). And after dehydration treatments, plants were re-watered 3 days, the leaves of WT remained wilted, while the OE lines grown normally (Fig. 3B).

### *CaNAC46* overexpression promotes the root growth of transgenic *Arabidopsis thaliana*

**Fig. 4:**
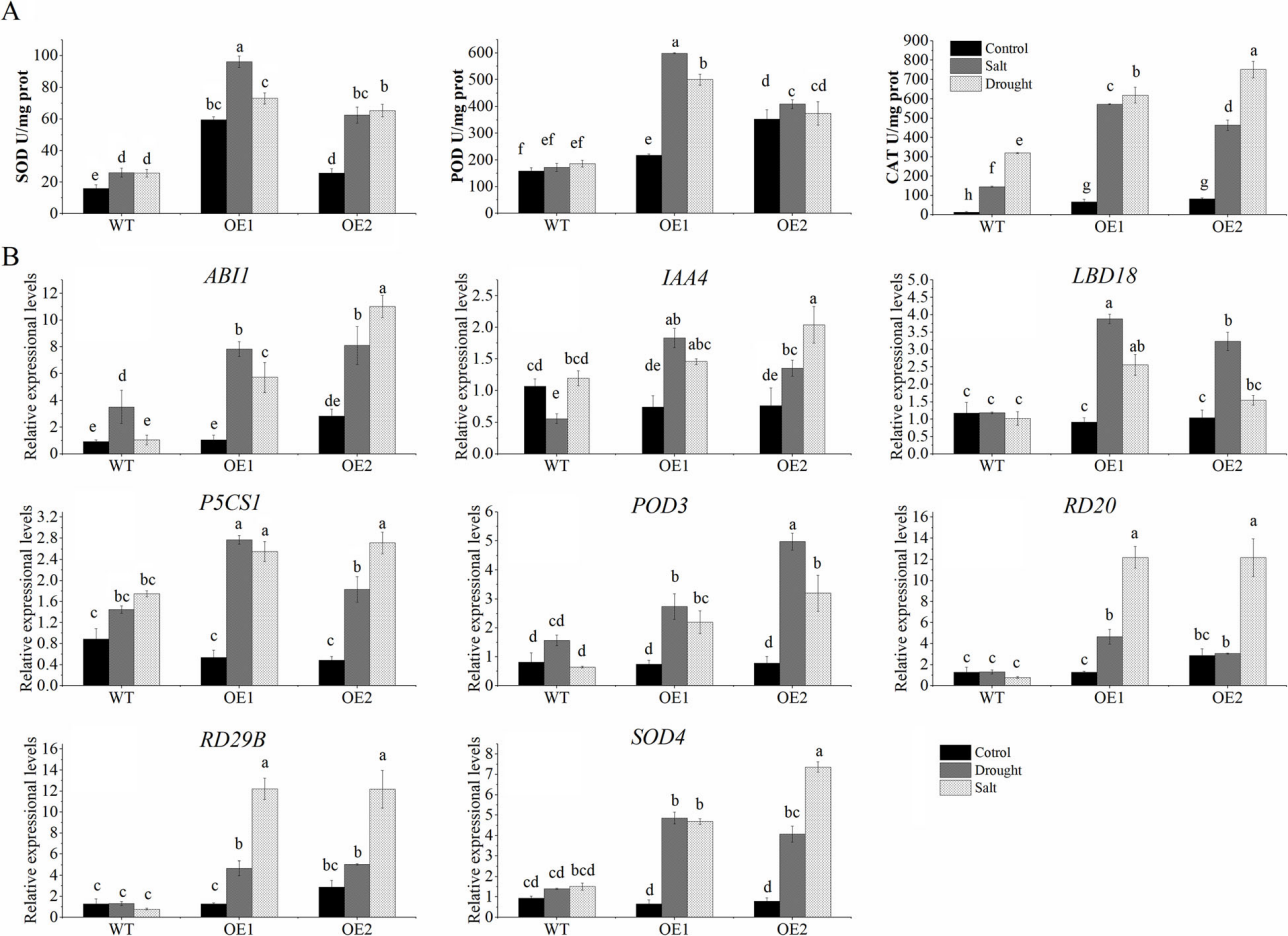
The physiological and stress-responsive gene expression analysis under stress treatments. **A** The activities of SOD, POD, and CAT were measured in the WT and *CaNAC46*-overexpressing plants under normal and stress conditions. **B** The expression levels of stress-responsive genes were in examined by qRT-PCR analyses the WT and CaNAC46-overexpressing plants before and after the abiotic stress treatments. Three independent biological experiments were performed. Superscript letters indicate significant differences (*P* < 0.05)

Plant root and shoot systems modify signaling and metabolic pathways to adapt to complex environmental changes and maintain normal growth and development [21]. A developed root system is important for improving plant stress resistance. In the current study, the NaCl treatment inhibited the lateral root development of WT seedlings. In contrast, the root elongation of two analyzed *CaNAC46*-overexpressing lines was only slightly inhibited, ultimately resulting in more lateral roots on the transgenic plants than on the WT plants (Fig. 3A and C). These observations implied that the overexpression of *CaNAC46* may enhance abiotic stress resistance by promoting root growth (Fig. 3). Moreover, the expression levels of *LBD18* and *IAA4*, which are involved in lateral root development, were significantly up-regulated in the *CaNAC46*-overexpressing plants under drought and saline conditions (Fig. 4B).

### *CaNAC46* overexpression decreases reactive oxygen species accumulation

High *CaNAC46* expression levels in the *CaNAC46*-overexpressing *A. thaliana* lines were confirmed by quantitative real-time PCR (qRT-PCR) (Additional file 3: Figure S2). Drought and salinity stresses usually induce the excessive accumulation of reactive oxygen species (ROS), which eventually leads to irreversible damages. We measured the SOD, POD, and CAT activities in the WT and *CaNAC46-*overexpressing plants under normal and stress conditions (Fig. 4A). There were no severe differences in the enzyme activities between the WT and transgenic plants under normal conditions. However, exposures to drought and salt stresses resulted in considerable increases in the SOD, POD, and CAT activities in both the WT and *CaNAC46-*overexpressing plants, but the increases were more pronounced in the *CaNAC46-*overexpressing plants.

### *CaNAC46* overexpression activates the expression of stress-related genes

To clarify the putative regulatory effect of CaNAC46 on abiotic stress tolerance, qRT-PCR analyses were completed to examine the expression patterns of stress-responsive genes (e.g., *ABI*, *P5CS1*, *RD29B*, and *RD20*) in the WT and *CaNAC46*-overexpressing plants before and after the abiotic stress treatments (Fig. 4B). We observed that a relatively low *CaNAC46* expression level impaired the water-deficit response of whole plants and detached leaves. In contrast, a relatively high *CaNAC46* expression level protected plant from the damages due to abiotic stresses.

### Silencing of *CaNAC46* decreases plant tolerance to high salt and drought conditions

**Fig. 5:**
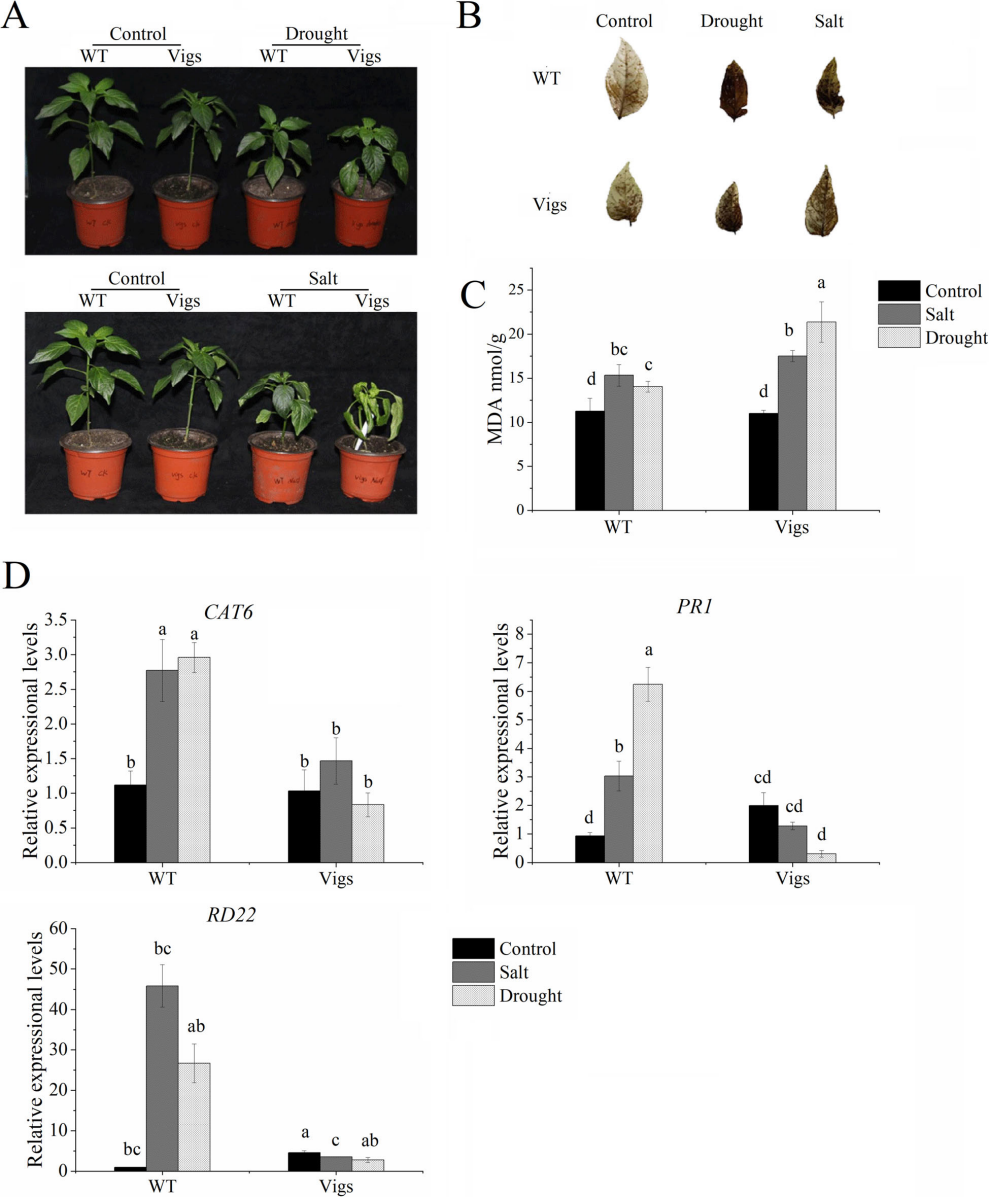
Suppression of *CaNAC46* leads to salt and drought sensitivity in pepper. The role of CaNAC46 for salt and drought sensitivity was assessed by VIGS. **A** The *TRV2-CaNAC46* and WT plants were subjected to drought stress by dehydration and salinity stress by watering with a 200 mM NaCl solution for 3 weeks. **B** DAB staining was in *TRV2-CaNAC46* and WT leaves than in WT leaves after for 3 weeks of the salt and drought treatments. **C** The MDA content of *TRV2-CaNAC46* and WT plants was measured under drought and salinity stresses. **D** The expression levels of stress-responsive genes were in examined by qRT-PCR analyses the WT and *TRV2-CaNAC46* plants before and after the abiotic stress treatments. Three independent biological experiments were performed. Superscript letters indicate significant differences (*P* < 0.05)

An analysis of the *CaNAC46* expression levels in the *CaNAC46*-silenced (*TRV2-CaNAC46*) and WT plants before the stress treatments confirmed that *CaNAC46* expression was significantly down-regulated in the *TRV2-CaNAC46* lines (Additional file 4: Figure S3). The *TRV2-CaNAC46* and WT plants were then subjected to drought stress (i.e., dehydrated) and salinity stress (i.e., treated with a 200 mM NaCl solution). The *TRV2-CaNAC46* lines showed a more weaker growth state than that of none-silenced lines under both normal and stress conditions (Fig. 5A). The cellular H_2_O_2_ level determined by diaminobenzidine (DAB) staining was higher in the *TRV2-CaNAC46* leaves than in the WT leaves after 3-week salt and drought treatments (Fig. 5B). Additionally, the malondialdehyde (MDA) content, which may be used as an indicator of lipid peroxidation, increased considerably in the *TRV2-CaNAC46* plants, but not in the WT plants, during the later stages of the drought and salinity treatments (Fig. 5C). Moreover, in the *TRV2-CaNAC46* plants, *CAT6* (encoding a catalase important for plant cell responses to stress), *PR1*, and *RD22* expression levels were down-regulated and the ROS-scavenging activity was low, which increased the sensitivity of these plants to saline and drought conditions (Fig. 5D).

## Discussion

Diverse stresses adversely affect the growth and productivity of dry pepper and represent the major yield-limiting factors. In plants, specific transcription factors enhance the resistance to external stresses [22]. We previously identified seven stress-responsive CaNAC46 transcription factors in *Capsicum* species. In the current study, we proved that the overexpression of *CaNAC46* increases the tolerance to various abiotic stresses, including drought and salinity.

The *CaNAC46* gene cloned from *C. annuum* ‘Qingnong No. 2’ consists of 921 bp that encode 306 amino acid residues. An analysis of the physicochemical properties revealed differences between CaNAC46 and NAC transcription factors from other plant species. This diversity may be relevant for future functional characterizations of NAC transcription factors. Additionally, CaNAC46 has three disordered amino acid regions, but their effects on the functions of the transcription factor remain to be determined. Ooka [8] classified NAC proteins into Groups I and II, with Group I divided into 14 subgroups. The NAC transcription factors in subgroups ATAF [23], AtNAC3 [24], and OsNAC3 [25] are involved in plant stress responses. The conserved sequences and phylogenetic relationships have been analyzed. The NAC transcription factors consist of a NAM subdomain with 160 amino acid residues as well as five other conserved subdomains (A, B, C, E, and D). In previous studies, the highly conserved subdomains A, C, and D were revealed to be important for NAC transcription factor functions. Subdomains C and D are mainly involved in DNA binding. The significance of subdomain A results from the insertion of the *dTph1* transposable element to form the *NAM* mutant [8]. Additionally, subdomains B and E vary among NAC proteins, suggesting they may be associated with the functional diversity of the NAC transcription factors [8]. Sequence comparisons with NAC family members confirmed that CaNAC46 belongs to the ATAF subfamily, and is most closely related to *A. thaliana* ATAF1.

Plant root system development is a relatively complex process, enabling plants to adapt to various environmental stresses. Salinity stress inhibits root meristem growth by decreasing auxin levels [26], whereas drought stress limits root growth and inhibits cytokinin production [27]. Root size and architecture influence the uptake of water and nutrients from the soil. In this study, *CaNAC46*-overexpressing plants produced longer roots and more lateral roots than WT plants following an exposure to drought and salinity stresses. Orthologs of *LBD18* in nonleguminous plants are required for lateral root development [28]. In *A. thaliana*, lateral root formation is regulated by indole-3-acetic acid (IAA) [29]. Our data indicated that *LBD18* and *IAA4* expression levels were up-regulated in the *CaNAC46*-overexpressing plants in response to drought and high salt conditions. Accordingly, CaNAC46 may promote lateral root formation and root elongation by up-regulating *LBD18* and *IAA4* expression to enhance the tolerance to drought and salinity stresses. The germination rate of *CaNAC46*-overexpressing lines was significantly lower than that of WT plants following the salt and drought treatments. These treatments also resulted in higher chlorophyll contents in the transgenic plants than in the WT controls.

In plants, proline is necessary for growth and development. Additionally, proline protects cells from damage by acting as a radical scavenger and an osmotic agent [15]. In the *TRV2-CaNAC46* lines, the down-regulated expression of *P5CS* increased the sensitivity of the plants to salt and drought stress conditions. A previous study indicated that RD26 is a transcriptional activator mediating ABA-inducible gene expression in plants subjected to abiotic stress [30]. The rice NAC family transcription factors OsNAP and OsNAC2 respond to abiotic stresses via the ABA pathway [31, 32]. The NAC transcription factors may minimize the accumulation of ROS and regulate the expression of stress-related genes under drought and high salt conditions [33]. We speculate that the observed enhanced tolerance to abiotic stresses is mainly due to the up-regulated expression of stress-responsive genes, including *RD20*, *RD29B*, *DREB*, and *RD22*. Following drought and salt treatments, *RD20* and *RD29B* expression levels were up-regulated in the *CaNAC46*-overexpressing plants and *DREB* and *RD22* expression levels were down-regulated in the *TRV2-CaNAC46* lines. The SOD, POD, and CAT activities were higher in the *CaNAC46*-overexpressing plants than in the control plants. Moreover, more MDA accumulated in the *TRV2-CaNAC46* plants than in the WT plants. The DAB staining results proved that ROS accumulation was greater in the *TRV2-CaNAC46* plants than in the control plants. The silencing of *CaNAC46* expression via virus-induced gene silencing (VIGS) resulted in oxidative damage, as indicated by the accumulation of H_2_O_2_. These results suggested that CaNAC46 can activate the expression of stress signaling-related genes in response to abiotic stresses. In the *CaNAC46*-overexpressing plants, the expression levels of *ABI*, *POD3*, *SOD2*, *P5CS1*, and other genes involved in the ROS-scavenging system were up-regulated to promote ROS scavenging and decrease stress-induced damages. The *TRV2-CaNAC46* plants had relatively weak ROS activities, making them more sensitive to salt and drought stresses than the WT plants. Furthermore, *CaNAC46* expression was correlated with CAT, POD, and SOD activities as well as with the transcription of most *SOD* and *POD* genes. Thus, silencing *CaNAC46* expression may be associated with increasing ROS accumulation and sensitivity to salt and drought stresses.

## Conclusion

Our results suggest that CaNAC46 localized in the nucleus helps regulate abiotic stress responses. In this study, *CaNAC46* expression was induced by high salinity, drought, heat, cold, ABA, SA, and MeJA treatments. The ectopic expression of *CaNAC46* in *A. thaliana* resulted in enhanced root formation and stronger development following drought and salt stress treatments. Moreover, the expression levels of several stress-responsive genes related to ROS scavenging and lateral root development were significantly up-regulated by drought and salt stress. Additionally, *CaNAC46-*silenced lines exhibited relatively weak growth and increased ROS production (i.e., more intense DAB staining) in response to salt and drought stress treatments. Furthermore, the expression of ROS-related genes was down-regulated in the *CaNAC46-*silenced lines. These findings imply that CaNAC46 may regulate salt and drought tolerance in pepper via its effects on ROS scavenging.

## Methods

### Plant materials, growth conditions, and treatments

*Capsicum annuum* L. var. ‘Qingnong No. 2’ was used as the plant material for various treatments and RNA isolation because of its tolerance to abiotic stresses. This cultivar was selected by researchers at Qingdao Agricultural University (Qingdao, China), the Qingdao Seed Station, and Dezhou Academy of Agricultural Sciences. Additionally, it was approved by the Shandong Variety Examination and Approval Committee in 2015 (deposition number: 2015–057-1). Pepper seeds were preserved at Qingdao Agricultural University (Qingdao, China). After a 3-day germination, seedlings were grown in a growth chamber at 28 °C with a 16-h light; 8-h dark cycle. All treatments were completed using 2-week-old seedlings. Regarding the simulated drought and saline conditions, seedling roots were treated with solutions containing 20% (w/v) PEG 6000 and 200 mM NaCl, respectively. For the cold and heat treatments, plants were transferred to growth chambers set to 4 and 37 °C, respectively. The phytohormone treatments involved immersing roots in an aqueous solution of 100 μM ABA, MeJA, or SA. Leaves were collected at 0, 2, 4, 8, 12, and 24 h after each treatment, immediately frozen in liquid nitrogen, ground to a powder, and then stored at − 80 °C.

### Isolation and sequence analysis of *CaNAC46*

Total RNA was extracted from leaves using the Plant Total RNA Kit (Yuanpinghao, Beijing, China). The RNA was then used as the template to synthesize cDNA with the PrimeScript RT Reagent Kit with gDNA Eraser (TAKARA, Dalian, China). The cDNA was diluted 20-fold for the subsequent TA cloning and qRT-PCR. The *CaNAC46* open reading frame was amplified by PCR using the following primers: forward: 5′-ATGATCAAAGGAATCGTTGGAAA-3′; reverse: 5′-CTAAGGTTTTTGCATGTATAGGA-3′. A multiple sequence alignment was completed with DNAMAN 6.0. The hydrophobicity, composition, and physicochemical properties of protein sequences were determined with the ExPASy online tools (http://www.expasy.org/). Additionally, a phylogenetic tree was constructed with MEGA 6.0.

### Quantitative real-time PCR

Gene expression levels were analyzed by qRT-PCR, which was completed with the reverse transcribed cDNA and the LightCycler® 480 SYBR Green I Master kit (TAKARA). The reaction solution comprised 7 μL ddH_2_O, 10 μL SYBR Green I Master mix, 1 μL Forward Primer (10 μmol/L), 1 μL Reverse Primer (10 μmol/L), and 1 μL cDNA. The PCR program was as follows: 95 °C for 3 min; 40 cycles of 95 °C for 5 s, 60 °C for 20 s, and 72 °C for 20 s. This program was followed by a melting curve analysis. All reactions were performed in triplicate. Relative gene expression levels were determined based on the 2^−ΔΔCt^ method. Primer sequences are listed in Table 1.

**Table 1:** Primer sequences used for real-time qRT-PCR amplification

Primer name	Primer sequence
At-acting-F	TTCCTCATGCCATCCTCCGTCTT
At-acting-R	CAGCGATACCTGAGAACATAGTG
AtABI1-F	GGATTTCA CCGGGATCAGATTGG
AtABI1-R	GTCACCGCAGTTAGCGACG AAG
AtRD20-F	TTAGCTCCGGTCACCAGTCA
AtABI1-R	TGTTCCATTCGGATGCTCTG
AtP5CS1-F	GTTTCCTCAGCCGCCGATTTTA
AtP5CS1-R	GGAAACGCCACGTGTGGAAC
AtRD29B-F	ATTCACCATCCAGAAGAAGAGCATC
AtRD29B-R	ACTTCTGGGTCTTGCTCGTCA
AtPOD3-F	CCAATCCGGAAACGGAAGTC
AtPOD3-R	TCTGCATACTTCTTGACGAG
AtSOD4-F	GAAGAACCTTGCTCCTTCCAG
AtSOD4-R	GATTGGCAGTTGTGTCAACAAC
AtIAA14-F	GCAGAGGAGGCAATGAGTAGTG
AtIAA14-R	GAGCATCCAGTCACCATCTTTG
AtLBD18-F	GTCGCTCACATCTTTGCTCTTC
AtLBD18-R	AGGTAGCTCTAGTGATGCCAAATG
CaNAC46-F	ATGATCAAAGGAATCGTTGGAAA
CaNAC46-R	CTAAGGTTTTTGCATGTATAGGA
CaPR1-F	ACTTGCAATTATGATCCACC
CaPR1-R	ACTCCAGTTACTGCACCAT
CaRD22-F	AGTAACTCTGGCAGTGGCAC
CaRD22-R	GTAGACTGGCGGCTTTCCTT
Ca-actin-F	GGTGACGAGGCTCAATCCAA
Ca-actin-R	CTCTGGAGCCACACGAAGTT
CaCAT6-F	TCTTGTTATTCGCGGGCCTT
CaCAT6-R	TGAAGGGGACAAACCAACCC

### Subcellular localization of CaNAC46

The *CaNAC46* coding sequence was amplified by PCR and inserted into the *Spe*I and *Asc*I sites of the modified pMDC83 vector with the ClonExpress II One Step Cloning Kit (Vazyme, Nanjing, China). After sequencing to confirm the accuracy of the inserted fragment, the recombinant plasmid and the empty pMDC83 vector (i.e., GFP alone) were introduced into tobacco cells according to an *Agrobacterium tumefaciens*-mediated method [34]. After a 24-h incubation in darkness, GFP fluorescence in transformed tobacco cells was observed with the FluoView™ FV300 confocal microscope (Olympus, Japan).

### Generation of *CaNAC46*-overexpressing transgenic *Arabidopsis thaliana* plants

The *CaNAC46* coding sequence was amplified by PCR and cloned into the *Xba*I sites of the pBI121 vector for the subsequent expression under the control of the cauliflower mosaic virus (CaMV) 35S promoter. The accuracy of the resulting construct was confirmed by sequencing, after which the pBI121-CaNAC46 recombinant plasmid was inserted into *A. thaliana* plants according to a floral dip method involving *A. tumefaciens* strain GV3101. Putative transgenic plants were screened on MS medium containing kanamycin and identified by PCR. Four T_3_ homozygous *CaNAC46*-overexpressing transgenic plants were selected for further analyses.

### Performance of *CaNAC46*-overexpressing *Arabidopsis thaliana* plants under saline and drought conditions

Homozygous *CaNAC46*-overexpressing lines from the T_3_ generation underwent a phenotypic analysis. The WT and transgenic lines were initially cultured on 1/2 MS agar medium at 22 °C with a 16-h light/8-h dark cycle for 7 days. The seedlings were then transferred to 1/2 MS agar medium supplemented with 200 mM NaCl or 10% PEG 6000. The seedlings were incubated for 1 week. Additionally, to investigate the drought tolerance of older plants, seedlings were grown in soil under normal conditions for 4 weeks, after which watering was withheld for 2 weeks and then resumed for 3 days. To evaluate the salt stress tolerance of older plants, WT and transgenic plants were grown in soil under normal conditions for 4 weeks, after which they were irrigated three times (4-day intervals) with a 200 mM NaCl solution.

### Malondialdehyde content and antioxidant enzyme activity measurements

The MDA contents as well as the SOD, POD, and CAT activities were quantified with commercial detection kits (Jiancheng Bioengineering Institute, Nanjing, China). The chlorophyll content was measured with a hand-held chlorophyll meter.

### Virus-induced gene silencing

A VIGS experiment was performed with pTRV1 and pTRV2 vectors. The *CaNAC46* sequence was inserted into the pTRV2 vector. The sequence to be silenced was determined using the SGN VIGS Tool (https://vigs.solgenomics.net/), and was amplified with the following primers (TRV-CaNAC46): forward: 5′-TTGGTGCAGCATTATTTGTGC-3′; reverse: 5′-ATTTTTGGTTTTATTTCATCCTCAA-3′. *Agrobacterium tumefaciens* cells containing pTRV1 were mixed with *A. tumefaciens* cells transformed with pTRV2-CaNAC46. The combined bacterial solution was infiltrated into the lower leaves of pepper plants. At 3 weeks post-infiltration, the silencing of *CaNAC46* was assessed via physiological measurements and histochemical assays.

### Statistical analysis

Bioassay results were evaluated with Origin and DPS. The significance of the differences in the data was determined with Student’s *t*-test (*P* < 0.05).

## Supplementary Information

**Additional file 1: Table S1.** A comparison of physical and chemical characterization of CaNAC46 transcription factors with its homolog NAC in other model plants.

**Additional file 2: Figure S1.** The germination rates of WT and *CaNAC46*-overexpressing plants under the simulated drought and salt conditions.

**Additional file 3: Figure S2.** The expression of *CaNAC46* gene in WT and OE plants by RT-PCR (1–4 represent WT plants; 5–8 represent OE plants).

**Additional file 4: Figure S3.** The expression of *CaNAC46* gene in WT and *TRV- CaNAC46* plants by RT-PCR (A, 1–4 represent WT plants, 5–8 represent *TRV- CaNAC46* plants) and by qRT-PCR (B, 1 represent WT, 2 represent *TRV- CaNAC46* plants).

## Data Availability

All data generated or analyzed during this study are included in this article (and its supplementary information files) or are available from the corresponding author on reasonable request. Sequence data from this article can be found in Arabidopsis Information Resource (https://www.arabidopsis.org/) under the following accession numbers: ATAF1 (At1g01720), ATAF2 (AT5G08790), ANAC036 (At2g17040), ANAC098 (At5g53950). The sequences of OsNAC005 (LOC_Os11g08210), OsNAC006 (LOC_Os01g66120), ONAC072 (LOC_Os09g32260) and ONAC022 (LOC_Os03g04070) are available in Genome Annotation Batch Download of Rice Genome Annotation Project (RGAP: http://rice.plantbiology.msu.edu/downloads_gad.shtml). In addition, the sequences of SlNAC053 (Solyc06g060230), SlNAC048 (Solyc05g055470), SlNAC067 (Solyc07g053590) and SlNAC091 (Solyc00g008000) are available in locus search of Sol Genomics Network (SGN: https://solgenomics.net/search/locus) database, and CaNAC52 (Capana05g002477), CaNAC71 (Capana07g002159), CaNAC101 (Capana12g002456), CaNAC20 (Capana02g000302), CaNAC46 (Capana05g000569) can be downloaded from the Pepper Genome Platform (PGP: http://peppergenome.snu.ac.kr/download.php). StNAC53 (CAC42087.1) is available in National Center for Biotechnology Information (NCBI, https://www.ncbi.nlm.nih.gov/).

## Abbreviations

ABA: Abscisic acid
ACC: 1-aminocyclopropane-1-carboxylic-acid
CaMV: Cauliflower mosaic virus
DAB: Diaminobezidine
IAA: Indole-3-acetic acid
LBD: Lateral organ boundaries domain
MDA: Malondialdehyde
MeJA: Methyl jasmonate
NAC: NAM, ATAF1/ATAF2, and CUC2
P5CS: Δ^1^-pyrroline-5-carboxylate synthetase
PEG: Polyethylene glycol
PLD: Phospholipase D
PR: Pathogenesis related
qRT-PCR: Quantitative reverse transcription-polymerase chain reaction
RD: Response to dehydration
ROS: Reactive oxygen species
SA: Salicylic acid
VIGS: Virus-induced gene silencing
WT: Wild type
