# Supplementary material for: The NAC-type transcription factor CaNAC46 regulates the salt and drought tolerance of transgenic Arabidopsis thaliana
Source: BMC Plant Biol. 2021 Jan 6;21:11. doi: 10.1186/s12870-020-02764-y (PMC7788707; doi:10.1186/s12870-020-02764-y)
Supplement: Supplementary file 1 — Additional file 1: Table S1. A comparison of physical and chemical characterization of CaNAC46 transcription factors with its homolog NAC in other model plants. [file 12870_2020_2764_MOESM1_ESM.docx]

Table S1 A comparison of physical and chemical characterization of CaNAC46 transcription factors with its homolog NAC in other model plants

| Plant | GenBank No. | No. of amino acid | Theoretical molecular weight | Theoretical pI | Percentage of basic amino acids | Percentage of acid amino acids | Percentage of aromatics amino acids | Percentage of aliphatic amino acids | Percentage of insolubility |
| --- | --- | --- | --- | --- | --- | --- | --- | --- | --- |
| *Capsicum annuum* | Capana05g000569 | 306 | 34941.42 | 6.96 | 15.5 | 12.8 | 11.4 | 62.84 | 49.6 |
|  |  |  |  |  |  |  |  |  |  |
| *Arabidopsis thaliana* | NP_171677.1 | 289 | 32922.33 | 6.15 | 13.9 | 13.2 | 15.4 | 60.38 | 54.52 |
| *Solanum lycopersicum* | NP_001234482 | 301 | 34835.24 | 7.62 | 15.3 | 12.6 | 12.3 | 58.7 | 38.46 |
| *Oryza sativa* | BAA89799 | 328 | 35375.61 | 5.47 | 13.4 | 13.7 | 7.6 | 60.21 | 46.72 |
| *Petunia hybrida* | ABS80935.11 | 303 | 34501.14 | 5.7 | 14.2 | 13.8 | 12.2 | 62.77 | 46.86 |
| *Nicotiana tabacum* | XM_009618363.2 | 319 | 36893.52 | 6.14 | 14.7 | 13.2 | 12.2 | 60.88 | 41.6 |
