# Supplementary figures and images for: The NAC-type transcription factor CaNAC46 regulates the salt and drought tolerance of transgenic Arabidopsis thaliana
Source: BMC Plant Biol. 2021 Jan 6;21:11. doi: 10.1186/s12870-020-02764-y (PMC7788707; doi:10.1186/s12870-020-02764-y)

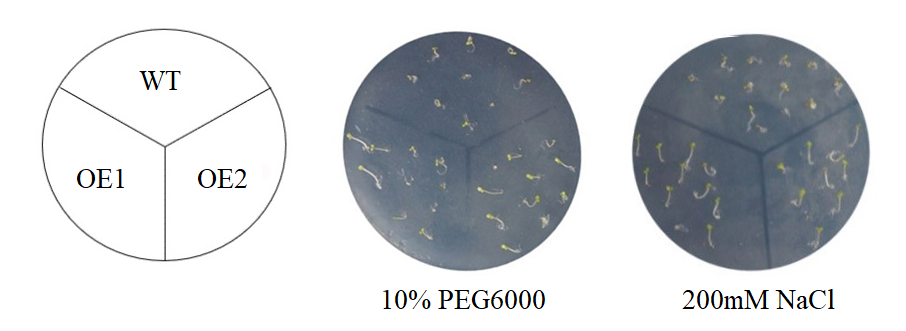

Supplement: Supplementary file 2 — Additional file 2: Figure S1. The germination rates of WT and CaNAC46-overexpressing plants under the simulated drought and salt conditions. [file 12870_2020_2764_MOESM2_ESM.tif]

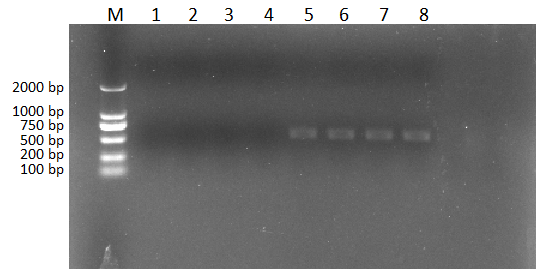

Supplement: Supplementary file 3 — Additional file 3: Figure S2. The expression of CaNAC46 gene in WT and OE plants by RT-PCR (1–4 represent WT plants; 5–8 represent OE plants). [file 12870_2020_2764_MOESM3_ESM.tif]

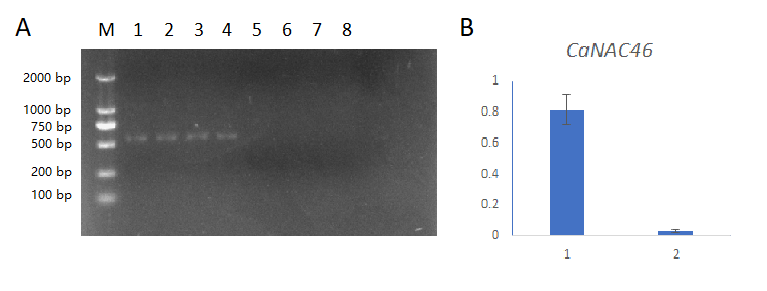

Supplement: Supplementary file 4 — Additional file 4: Figure S3. The expression of CaNAC46 gene in WT and TRV- CaNAC46 plants by RT-PCR (A, 1–4 represent WT plants, 5–8 represent TRV- CaNAC46 plants) and by qRT-PCR (B, 1 represent WT, 2 represent TRV- CaNAC46 plants). [file 12870_2020_2764_MOESM4_ESM.tif]
